# Prevalence of Depression, Anxiety and Post-Traumatic Stress Disorder (PTSD) After Acute Myocardial Infarction: A Systematic Review and Meta-Analysis †

**DOI:** 10.3390/jcm14061786

**Published:** 2025-03-07

**Authors:** Ray Junrui Chong, Yunrui Hao, Emily Wei Qi Tan, Grace Jing Le Mok, Ching-Hui Sia, Jamie Sin Ying Ho, Mark Yan Yee Chan, Andrew Fu Wah Ho

**Affiliations:** 1 Yong Loo Lin School of Medicine, National University of Singapore, Singapore 117597, Singapore; e1080789@u.nus.edu; 2 Lee Kong Chian School of Medicine, Nanyang Technological University, Singapore 639798, Singapore; wei.qi.tan@mohh.com.sg (E.W.Q.T.); gmok003@e.ntu.edu.sg (G.J.L.M.); 3 Department of Cardiology, National University Heart Centre Singapore, Singapore 119074, Singapore; ching_hui_sia@nuhs.edu.sg (C.-H.S.); mdccyym@nus.edu.sg (M.Y.Y.C.); 4 Department of Medicine, National University Hospital, Singapore 119074, Singapore; sinying.ho@mohh.com.sg; 5 Department of Emergency Medicine, Singapore General Hospital, Singapore 169608, Singapore; 6 Pre-Hospital & Emergency Research Centre, Duke-National University of Singapore Medical School, Singapore 169857, Singapore; 7 Centre for Population Health Research and Implementation, SingHealth Regional Health System, Singapore 117549, Singapore

**Keywords:** myocardial infarction, AMI, depression, anxiety, post-traumatic stress disorder, PTSD, psychiatrist, DSM, diagnostic and statistics manual

## Abstract

**Background**: Mental illnesses following an acute myocardial infarction (AMI) are a growing concern, as they are associated with worse outcomes for AMI patients. Our understanding of the prevalence of mental illnesses after an AMI is incomplete, as most studies investigate depression while overlooking other conditions like anxiety and PTSD. Existing studies often rely on patient-reported questionnaires for mental illness diagnoses, a method that can be subjective. To address this, we conducted a systematic review and meta-analysis to determine the prevalence and risk factors of depression, anxiety, and PTSD after AMI, including only studies with formal mental illness diagnoses. **Methods**: Searches in MEDLINE, EMBASE, and PsycINFO up to 23 January 2025 identified 23 qualifying studies that assessed the prevalence of depression, anxiety, and PTSD after AMI, with cases defined exclusively by formal diagnoses established through psychiatrist-administered structured interviews according to the *Diagnostic and Statistical Manual for Mental Disorders* (DSM) criteria (versions III to V). For each outcome, the pooled prevalence was estimated using meta-analyses of proportions with random-effects models. If significant heterogeneity was detected, subgroup analyses and meta-regression were performed to explore the factors contributing to this heterogeneity. **Results**: A total of 25 studies were included in the meta-analysis. Among the 20 studies included, the pooled prevalence of depression after AMI was 23.58% (95% CI: 22.86%; 24.32%). When stratified by time since AMI, the prevalence was 19.46% (95% CI: 15.47%; 24.19%) for those assessed within 3 months and 14.87% (95% CI: 9.55%; 22.43%) for those assessed after 3 months. The pooled prevalence of anxiety (seven studies) and PTSD (three studies) was 11.96% (95% CI: 6.15; 21.96%) and 10.26% (95% CI: 5.49%; 18.36%), respectively. Further pooled prevalence subgroup analysis of depression and anxiety revealed significantly higher rates in the female sex (29.89%, 95% CI: 21.85; 39.41%), in those with hypertension (25.01%, 95% CI: 21.68; 28.67%), diabetes (25.01%, 95% CI: 21.68; 28.67%), or hyperlipidemia (28.96% 95% CI: 23.44; 35.17%), and in smokers (25.23%., 95% CI: 19.48; 32.00%), while the pooled prevalence of depression is higher in unmarried (35.44%, 95% CI: 19.61; 55.26%) than married individuals (28.63%, 95% CI: 18.67; 41.20%) and in those with a history of depression (57.41%, 95% CI: 31.47; 78.92%). The results of the meta-regression indicated that a prior history of depression was a significant predictor of depression prevalence (*p* = 0.0035, regression coefficient 1.54). **Conclusions**: The prevalence of mental illnesses, including depression, anxiety, and PTSD, is notable following an AMI. Identified risk factors encompass female sex, hypertension, diabetes mellitus, hyperlipidemia, smoking, a history of depressive illness, and social context.

## 1. Introduction

Cardiovascular disease (CVD) represents the leading cause of global mortality, responsible for 32% of deaths, of which 85% are caused by acute myocardial infarction (AMI) and stroke [1]. Despite advancements in AMI treatment and prognosis, 30-day and 1-year mortality rates remain at 5–8% and 10–12%, respectively [2]. Further research is needed to develop strategies to improve patient outcomes

Recent years have witnessed a rise in diagnosed mental health disorders (depression, anxiety, stress) and their association with both CVD development and poorer AMI outcomes [3]. The ENRICHD study revealed a 2.22-fold increased risk of earlier hospitalization with a higher frequency of hospitalizations and ED visits in post-MI patients with depression compared to non-depressed patients [4]. Post-MI depression, as shown in a meta-analysis of 29 studies (*n* = 16,889), significantly elevates the risk of adverse outcomes, with a 2.25 times higher risk of all-cause mortality, a 2.71 times higher risk of cardiac-related death, and a 1.59 times higher risk of experiencing new cardiac events [5]. Similarly, anxiety and post-traumatic stress disorder (PTSD) are associated with poorer outcomes such as increased readmission and cardiovascular mortality in post-MI patients [6,7].

While the three conditions—depression, anxiety, and PTSD—share overlapping symptoms, they differ in triggers, presentation, and clinical implications.

Depression is marked by persistent low mood, loss of interest, and cognitive difficulties, often leading to social withdrawal and impaired functioning. Individuals with anxiety disorders, like generalized anxiety disorder and panic disorder, experience excessive worry and heightened fear, often accompanied by physical symptoms like palpitations and restlessness. These symptoms can negatively affect their relationships and daily lives. PTSD, triggered by trauma, is characterized by intrusive memories, hyperarousal, and avoidance behaviors, often disrupting emotional stability and increasing the risk of substance abuse.

Accurate characterization of mental health disorder prevalence post-AMI is necessary due to variable reporting in post-AMI recovery. A meta-analysis in 2019 found that the pooled prevalence of depression in MI patients was 28.7% among 19 eligible studies, but with significant heterogeneity (I^2^ = 98.4%), ranging from 9.17% to 65.88% [8]. The meta-analysis by Wen et al. found that the prevalence of anxiety ranged from 5.5% to 58.2% [7]. A meta-analysis in 2012 found that the prevalence of PTSD in acute coronary syndrome patients ranged widely from 0 to 32% [9]. The large variation in prevalence may be contributed to by variations in diagnostic methods, the inclusion of studies using patient-reported questionnaires, and patients with pre-existing mental health disorders.

Recognizing the profound impact of psychiatric conditions in post-MI patients is a crucial step in providing comprehensive and patient-centered care. The objective of this meta-analysis is to determine the prevalence of depression, anxiety, and PTSD based on formal diagnosis through psychiatrist-administered structured interviews among post-MI patients. This article is a revised and expanded version of a paper entitled *Prevalence of depression, anxiety and post-traumatic stress disorder (PTSD) after acute myocardial infarction: a systematic review and meta-analysis*, which was presented at ESC Congress 2024, London.

## 2. Methods

### 2.1. Ethics Approval

This study analyzed published data; hence, ethics approval was not necessary.

### 2.2. Patient and Public Involvement

Patients and the public were not involved in our study.

### 2.3. Search Strategy

This review is registered on PROSPERO (CRD42024568992) and is reported in accordance with the 2020 Preferred Reporting Items for Systematic Reviews and Meta-Analyses (PRISMA) guidelines [9]. A comprehensive search of the existing literature was performed in MEDLINE, EMBASE and PsycINFO from database inception—MEDLINE from 1946, EMBASE from 1947, and PsycINFO from 1967—to January 2025 using keywords related to “Major Depression”, “Anxiety”, “Post-Traumatic Stress Disorder”, and “Myocardial Infarction”. The full search strategy was developed in collaboration with a medical information specialist at the Medical Libraries, National University of Singapore, and is available in the Supplementary Materials.

### 2.4. Study Selection

Researchers were paired (RCJ with HYR, TWQE with GMJL), and each individual in the pair independently conducted the selection of studies, after which conflicts were resolved by a third researcher by adhering strictly to the inclusion and exclusion criteria. Inclusion criteria were as follows: (a) cross-sectional, prospective or retrospective studies on adults (>18 year old) with established AMI; (b) patients with a formal diagnosis of depression, anxiety, or PTSD based on a psychiatrist-administered structured interview following the *Diagnostic and Statistical Manual for Mental Disorders* (DSM) or the International Classification of Diseases (ICD) criteria [10]; (c) studies that reported the prevalence of post-AMI depression, anxiety, and/or PTSD. Studies were limited to those published in peer-reviewed journals in English, or with accompanying English translations. Data duplication was avoided by collating studies originating from the same center(s) with overlapping time periods, and from these studies, only the study with the longest follow-up was selected.

Exclusion criteria were as follows: (a) studies that lacked primary data (e.g., narrative reviews, systematic reviews); (b) reported on a sample size less than 100; (c) included patients with a diagnosis of mental health disorders using patient-reported questionnaires.

### 2.5. Data Collection

Two researchers extracted data from each selected article: author, publication year, country of population, study design, sample source (hospital-based/population-based), sample size, mean/median age, percentage of male participants, timing of depression/anxiety assessment, prevalence of a psychiatric condition at each timing, and the assessment tool used to determine the presence of a psychiatric condition. Additional information on demographics (percentage of married participants, percentage of participants who smoke, education level, percentage of employed participants) and medical history (percentage of participants with diabetes mellitus, percentage of patients with hypertension, percentage of anterior MI, percentage of inferior MI, median/mean Killip class, previous history of MI) was extracted when available.

### 2.6. Assessment of Depression, Anxiety, and PTSD

The gold standard for the diagnosis of psychiatric conditions is structured interviews with qualified healthcare professionals, such as the Composite International Diagnostic Interview (CIDI) and Mini International Neuropsychiatric Interview (MINI). Patients should meet established criteria (ICD-9, ICD-10, or DSM III or later diagnostic criteria.

### 2.7. Quality Assessment of Studies

The quality of all 23 selected studies was assessed using the Newcastle–Ottawa Scale for non-randomized studies by two authors independently. The checklist consists of a ’star system’ which assesses the risk of bias of the study from three broad perspectives: the selection of the study groups; the comparability of the groups; and the ascertainment of either the exposure or outcome of interest for case–control or cohort studies. Each assessed study is then graded out of a total score of 9 and further categorized into high (fewer than 4), moderate (4 to 6), and low (7 to 9) risk of bias [11]. In total, 23 papers were assessed for their risk of bias.

### 2.8. Statistical Analysis

The pooled prevalence of depression, anxiety, and PTSD among patients with AMI was calculated using a random-effects model by the Freeman–Tukey double arcsine method, and each corresponding 95% confidence interval (CI) was calculated. The heterogeneity across studies was quantified by the I^2^ statistic, with its values of 25%, 50%, and 75% indicating low, moderate, and high heterogeneity, respectively.

In the presence of significant heterogeneity, meta-regression analyses were performed with the mean age of participants, percentage of male participants, percentage of participants with first-time AMI, and quality assessment score as covariates. Subgroup analyses were performed to investigate the pooled prevalence of depression, anxiety, and PTSD in AMI patients. The following factors were included in these analyses where data were available: region, study quality, depression identification tool, sex, race, marital status, history of previous MI, anterior MI, Killip class, current smoking status, hypertension, hyperlipidemia, and diabetes mellitus. Chi-square tests assessed subgroup differences in pooled depression prevalence, with a *p*-value of less than 0.05 considered statistically significant.

Sensitivity analysis was performed by leave-one-out analysis and removing the studies with relatively low quality. Publication bias was assessed using funnel plots and verified by Egger’s and Begg’s tests. Statistical analyses were conducted using R statistical software, version 3.4.1 (R Core Team, 2021).

## 3. Results

**Figure 1:**
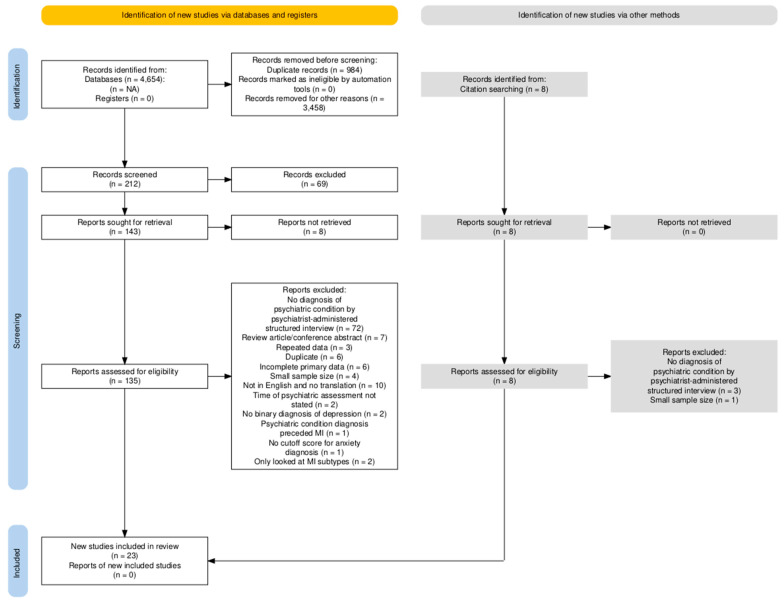
PRISM flowchart of study selection.

A total of 4647 records were identified through the search strategy, with 23 studies included (Figure 1: flow diagram). For each study, baseline characteristics of the population and other comorbidities were collected (Supplementary Table S1). Out of these 23 studies, 20, 7, and 3 were used in the meta-analysis of the prevalence of depression, anxiety, and PTSD, respectively. These 23 studies were conducted in eight different countries: India, Turkey, Taiwan, USA, the Netherlands, Canada, Austria, and Switzerland. All studies were hospital-based, including both inpatient and outpatient settings, and psychiatric conditions were diagnosed based on a structured interview with a psychiatrist according to DSM III-V criteria. The mean age of participants ranged from 51.96 to 74.5 years, and the proportion of male participants ranged from 54 to 91.3%. Three studies included exclusively patients with first-time myocardial infarction [12] (Supplementary Table S1). The quality assessment of all eligible studies showed a low to moderate risk of bias. There were 21 low-risk studies and 2 moderate-risk studies. The cut-off scores for low risk and moderate risk are <6 and <3, respectively (Supplementary Table S2).

### 3.1. Pooled Prevalence of Post-AMI Depression

**Figure 2:**
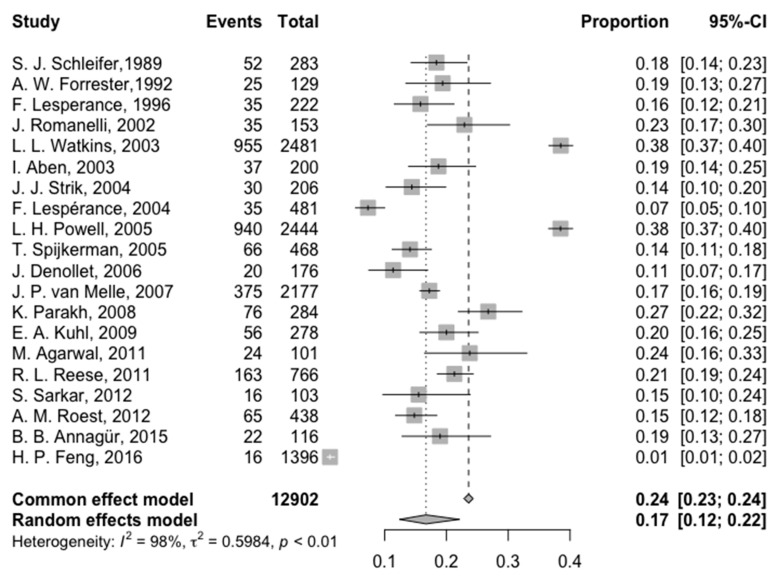
Forest plot of the meta-analysis of prevalence of post-AMI depression [4,13,14,15,16,17,18,19,20,21,22,23,24,25,26,27,28,29,30,31].

The pooled prevalence of depression post-AMI across the 20 studies (3043 patients of 12,902 participants developed depression) was 23.58% (95% CI: 22.86%; 24.32%) (Figure 2). Significant heterogeneity (I^2^ = 97.8%) was observed, with individual study prevalence ranging from 1.15% (Taiwan, ICD-9 criteria) to 38.5% (USA, ICD-10 criteria).

**Figure 3:**
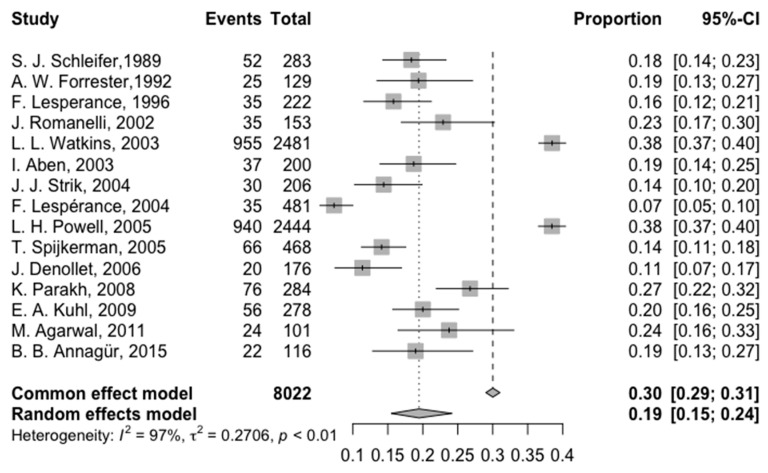
Forest plot of the prevalence of depression at less than 3 months of follow-up [13,14,15,16,17,18,19,20,21,22,23,25,26,27,30].

The pooled depression prevalence in AMI patients was analyzed at ≤3- and >3-month follow-ups (*n* = 20 studies). At ≤3 months (*n* = 15), the pooled prevalence was 19.46% (95% CI: 15.47%; 24.19%), with significant heterogeneity (I^2^ = 96.7%) (Figure 3). The lowest prevalence among these studies was 5.08% and the highest was 38.49%.

**Figure 4:**
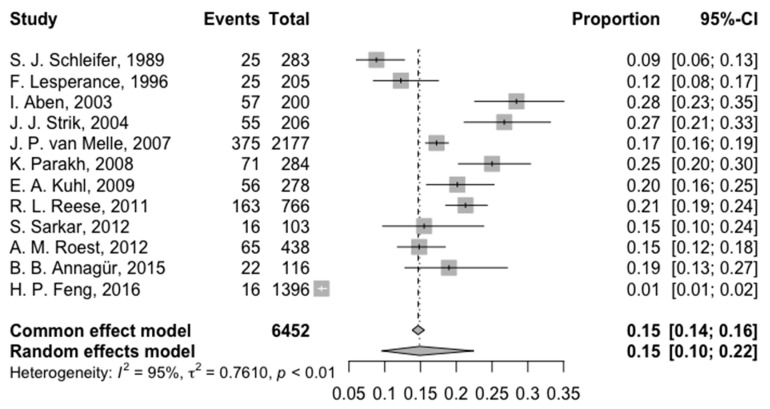
Forest plot of the prevalence of depression at more than 3 months of follow-up [4,13,15,18,19,24,25,26,28,29,30,31].

At >3 months, pooled depression prevalence was 14.87% (95% CI: 9.55%; 22.43%), with significant heterogeneity (I^2^ = 94.5% [92.1%; 96.2%]) (Figure 4). Prevalence ranged from 1.15% to 32.21%.

### 3.2. Meta-Regression and Subgroup Analysis of Prevalence of Depression

History of depression is a significant predictor of depression among post-AMI patients (*p* = 0.0035, regression coefficient 1.54), while hyperlipidemia (*p* = 0.7617), diabetes mellitus (*p* = 0.9059), and sex (*p* = 0.3609) were not significant moderators. Data limitations precluded meta-regression for hypertension and smoking.

Subgroup analyses of sex, smoking status, living status, marital status, medical conditions (hypertension, hyperlipidemia, DM), and AMI presentation (Killip class, anterior, first-time) were performed with the Chi-squared test of independence (Supplementary Table S3). The prevalence of depression in females was 29.89% (95% CI: 21.85; 39.41%), which was higher than that in males at 22.06% (95% CI: 17.01–28.10%), and the *p*-value was less than 0.05. The prevalence of depression among those with hypertension (25.01%, 95% CI: 21.68; 28.67%), hyperlipidemia (28.96%, 95% CI: 23.44; 35.17%), and diabetes mellitus (34.29%, 95% CI: 28.11; 41.04%) was significantly higher than those without the respective conditions (19.45%, 19.69%, 28.33%). Individuals with a Killip class > 1 (34.48%, 95% CI: 23.76; 47.06%) and smokers (25.23%, 95% CI: 19.48; 32.00%) had a higher prevalence of depression than their counterparts as well. Social set-up also had a significant impact on the prevalence of depression, with higher prevalence in unmarried participants (35.44%, 95% CI: 19.61; 55.26%). The prevalence of depression was similar in participants with anterior MI (43.75%, 95% CI: 21.72; 68.54%) and without anterior MI (31.07%, 95% CI: 18.07; 47.95%).

### 3.3. Prevalence of Anxiety Among Patients with MI

**Figure 5:**
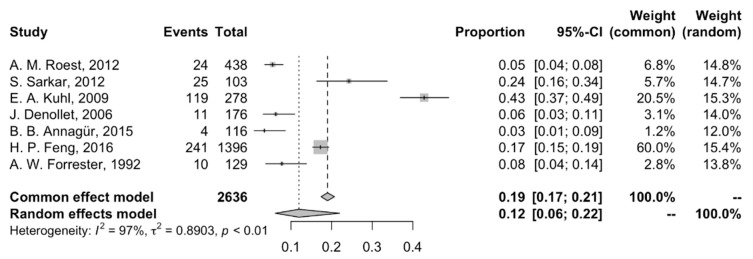
Forest plot of the meta-analysis of prevalence of post-AMI anxiety [14,23,26,28,29,30,31].

Among the seven eligible studies assessing the prevalence of anxiety in patients post-MI, the pooled prevalence was 11.96% (95% CI: 6.15; 21.96%), and significant heterogeneity was observed (I^2^ = 97%) (Figure 5). The individual study prevalence of anxiety ranged from 3.45% (Turkey, DSM-IV criteria) to 42.81% (USA, DSM-III-R criteria) [32].

Subgroup prevalence analysis of anxiety in MI patients revealed a significantly higher prevalence amongst female participants, smokers, and those with hypertension, hyperlipidemia, a DM Killip class more than 1, and a history of depression (all with *p*-value < 0.05) (Supplementary Table S4). Notably, all subgroups displayed high heterogeneity.

### 3.4. Pooled Prevalence of PTSD Among Patients with MI

**Figure 6:**
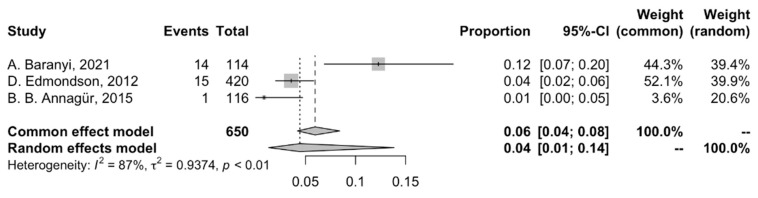
Forest plot of the meta-analysis of prevalence of post-AMI PTSD [30,33,34].

The pooled prevalence of PTSD in patients with MI in the three eligible studies was 4.43% (95% CI: 1.32; 13.84%), with significant heterogeneity (I^2^ = 87.3%) (Figure 6). The prevalence of PTSD ranged from 0.86% (Turkey, DSM-IV Structured Clinical Interview) to 12.28% (Austria, DSM-V Clinician-Administered PTSD Scale).

**Figure 7:**
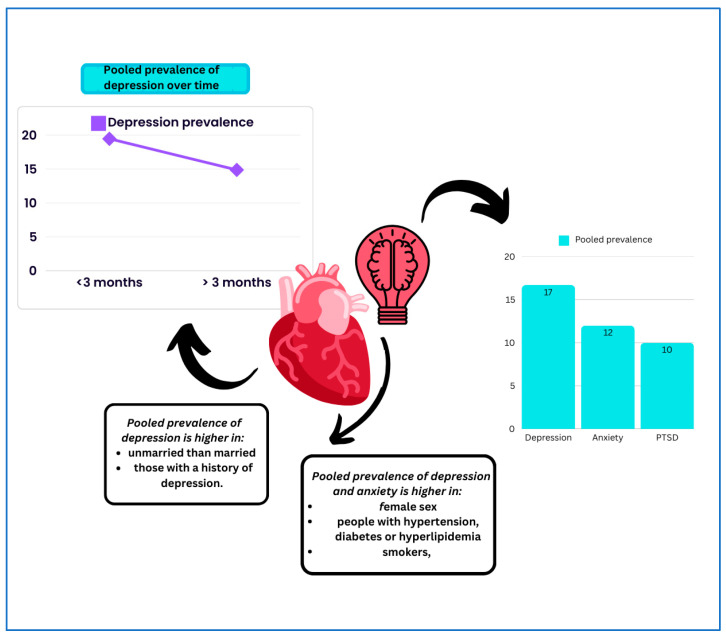
Visual summary of the study findings.

The overall findings of this review are summarized in Figure 7.

### 3.5. Sensitivity Analysis and Publication Bias

**Figure 8:**
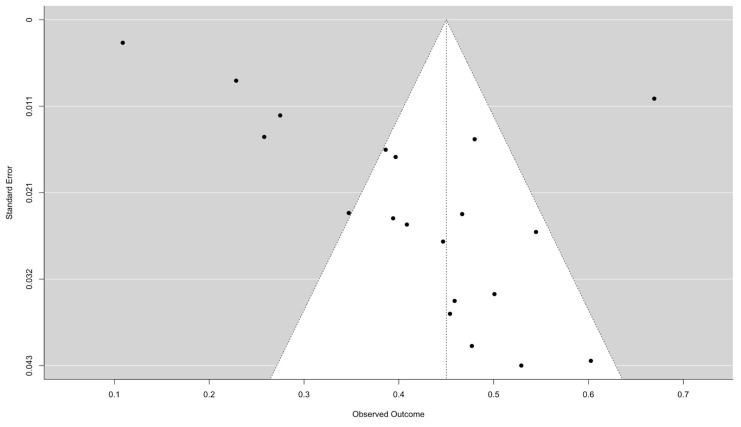
Funnel plot of the eligible studies.

After excluding studies with moderate quality, the pooled prevalence of depression increased from 20.25% to 26.66% (95% CI: 19.75; 34.92), the pooled prevalence of anxiety increased from 13.66% (95% CI: 6.67; 25.94%) to 18.61% (95% CI: 5.90; 45.44%), and the pooled prevalence of PTSD increased from 10.26% (95% CI: 5.49; 18.36%) to 10.40% (95% CI: 5.47; 18.87%). Egger’s test result was t = −8.51 (*p* = 0.000106), and the Begg’s test result was z = −0.580 (*p* = 0.559), indicating no evidence of publication bias. The funnel plot was symmetrical in alignment with these results (Figure 8).

## 4. Discussion

The main findings of this meta-analysis were as follows: (1) the prevalence of post-MI depression, anxiety, and PTSD was 16.7%, 12.0%, and 4.4%, respectively; (2) depression was present in around 15% of patients beyond 3 months post-AMI; and (3) factors associated with the development of mental health disorders include sex, symptomatic burden, and presence of other comorbidities.

Post-AMI depression is prevalent, impacts quality of life, and increases mortality. A previous meta-analysis by Thombs et al. (2004) reported a pooled prevalence of 19.8% using structured interviews, ranging from 16 to 45%, but study heterogeneity was not explored [12]. In the 2018 meta-analysis by Feng et al., the pooled prevalence of depression was 28.7%, ranging from 9.17 to 65.88%, with high heterogeneity among 19 studies [8]. The latter study included depression identified from both structured interviews and self-report questionnaires. Structured interviews are the gold standard for the diagnosis of psychiatric disorders, and the correlation between structured interviews and self-reported diagnostic measures for major depression and anxiety showed only moderate agreement, which varied by cohorts [32]. Using only structured interviews for diagnosis (gold standard), our meta-analysis finds a range of prevalence from 1.15% to 38.49% with a pooled prevalence of 20.25% (95% CI: 12.83%; 30.47%). This is lower than Feng et al.’s estimate, likely due to stricter assessment methods, but heterogeneity remains high, suggesting that true prevalence may vary in different study populations.

Our meta-analysis found that while around 20% of patients had depression within 3 months of AMI, it may persist beyond 3 months. We found that 15% had depression in studies beyond 3 months, similar to previously reported figures. For example, Schleifer et al. (1991) found a prevalence of 17% for major depression 8–10 days post-AMI, and 14% at 4 months [35]. Lauzon et al. (2003), who used the symptoms-based Beck Depression Inventory (BDI) questionnaire, showed that 39% of patients had depression (BDI < 10) at 30 days, 39% at 6 months, and 30% at 1 year [36]. A higher depression prevalence on the BDI may be attributed to overdiagnosis associated with self-administered questionnaires, reflected in another study where nurse-administered SCID and BDI only had 13.5% agreement in the post-AMI setting [37].

Similar to depression, AMI is associated with anxiety development, although research on anxiety is less extensive. In Aw et al.’s (2021) meta-analysis, the pooled prevalence of anxiety in AMI patients was 9.1%, but this included diagnosis by self-administered questionnaires [38]. Our meta-analysis, using structured interviews only, reports a prevalence of anxiety ranging from 3.45% to 42.81% with a pooled prevalence of 11.96% (95% CI: 6.15; 21.96%), but inter-study heterogeneity remains high.

The link between AMI and depression or anxiety is likely multifaceted. AMI activates the hypothalamic–pituitary–adrenal (HPA) axis, and patients with depression for more than 3 months showed lower mean morning and evening serum cortisol concentrations [39]. Ischemic injury post-AMI triggers an inflammatory cascade, with elevated pro-inflammatory cytokines (IL-1β, IL-6, TNF-α, sICAM-1), which have been linked to depression and anxiety [40,41]. Chronic inflammation affects neurotransmitter metabolism and reduces brain-derived neurotrophic factor (BDNF) levels, impairing neuroplasticity and mood regulation [42]. The effect of anti-inflammatory medications on post-MI depression and anxiety requires further investigation.

There was consistently high inter-study heterogeneity in the published meta-analyses of the prevalence of both anxiety and depression post-AMI [8,12,38], suggesting that patient, environmental, social, and clinical factors influence risk. Sex emerged as a significant factor, where females exhibited a higher prevalence of depression and anxiety [8,43]. This was also seen in patients post-stroke, where females were more commonly depressed in a meta-analysis of 47 studies [43]. Possible hypotheses include physiological changes (e.g., menopause), social factors, differences in economic status and coping resources, opportunity structures for help-seeking behavior [44], and greater tendencies towards ruminative coping styles [45]. However, the observed sex disparity might reflect the normalization of help-seeking behavior amongst women as compared to men [46].

There may also exist other important but often overlooked factors that lead to increased post-AMI depression rates and suboptimal treatment. Patients with pre-existing psychiatric conditions are also at a higher risk for cardiovascular disease (CVD), and this risk can increase after an AMI [47]. Their atypical symptoms, such as jaw or back discomfort, often lead to misdiagnosis and delayed care, increasing psychological distress. Additionally, healthcare biases may result in symptoms being misattributed to anxiety, further delaying treatment and exacerbating mental health risks [48].

Furthermore, the female gender is severely underrepresented in cardiovascular research, limiting gender-specific treatment strategies, while socio-cultural factors, such as caregiving responsibilities, contribute to delayed care-seeking and heightened stress. Addressing these disparities requires improved symptom recognition, reduced diagnostic bias, increased female representation in research, and consideration of social factors in treatment planning [48].

Comorbidities such as hypertension, hyperlipidemia, DM, and smoking may also be risk factors for depression and anxiety. In the general population, DM is associated with a 2–3 times higher risk of depression, and up to 40% of patients with DM have anxiety [49]. Potential mechanisms include (i) shared risk factors such as low socioeconomic status, and lifestyle factors such as lack of exercise, poor sleep, and diet; and (ii) biological pathways such as overactivation of the HPA axis leading to mood instability [50]. Smoking also increases the risk of depression, with evidence suggesting that cessation improves mood [51]. While short-term nicotine use relieves stress, long-term use leads to addiction and dependence, increasing vulnerability to depression and anxiety [52]. A bidirectional relationship is often observed where mental health illnesses influence physical conditions, in this case the development of cardiovascular risk factors, by reducing adherence to secondary prevention or lifestyle modifications (e.g., low-fat or -sugar diet, exercise) and medication compliance [53].

Social factors may also influence the development of psychiatric disorders post-AMI. Unmarried patients exhibited a trend towards higher post-AMI depression rates, aligning with earlier studies demonstrating that social isolation (living alone or unpartnered) is linked to poor mental health. This can manifest as depression and anxiety and is itself an important contributor to hospital readmission in heart disease patients [54]. Psychosocial interventions such as group therapy post-AMI may reduce social isolation, although clinical trials did not demonstrate any significant differences in survival [55]. Further research is needed to evaluate the efficacy of social interventions in post-AMI patients, especially for those experiencing social isolation.

In terms of PTSD, albeit a known psychiatric complication of AMI, its true prevalence is debated due to over- or underdiagnosis on self-reported questionnaires or disclosure reluctance, respectively. A 2012 meta-analysis, incorporating both structured interviews and self-report questionnaires, estimated PTSD prevalence at 12% (0–32%) [9]. However, our stricter meta-analysis, limited to structured psychiatric interviews, found a pooled prevalence of ~4% (range: 1–12%). Limited data precluded meta-regressions or subgroup analysis, although previous researchers have posited that social determinants like female sex, young age, and low socioeconomic status may be significant PTSD risk factors after major cardiac events [56]. Future studies are required to analyze PTSD prevalence across demographics, to gain a deeper understanding of post-AMI PTSD risk factors, and to develop preventative interventions.

The findings on the longitudinal progression of mental illnesses post-AMI in the present review are immensely valuable, as such psychiatric complications correlate closely with poor long-term outcomes such as heightened 4-month mortality [57], increased 30-day readmissions (HR 1.09–1.56) [58], a two-fold increase in cardiac events over 10 years, and impaired return to pre-AMI activities, importantly returning to work within 3 months post-AMI, and medication affordability issues. All these translate into compromised quality of life for such patients [59].

Given the established link of mental health disorders like depression and anxiety with cardiovascular diseases, international guidelines such as the 2023 American Heart Association guidelines endorse targeted discussions and mental health screening for clinician assessment and potential referral (Class 2a) [60]. Our meta-analysis informs clinicians that around 1 in 6 patients post-AMI may develop depression, 1 in 8 for anxiety, and 1 in 25 for PTSD. Female patients, patients with more severe AMI presentation (Killip class), and those with more comorbidities (hypertension, hyperlipidemia, DM, and smoking) are at heightened risk, warranting prioritized mental health screening in high-risk subgroups to significantly modify long-term post-AMI outcomes.

Management-wise, mental health care should be integrated into cardiac rehabilitation programs, ensuring a holistic approach to patient recovery. This could start with the inclusion of mental health professionals as part of the multidisciplinary team, as well as introducing regular mental health screenings, psychological counseling, and stress management workshops into the program. Abbreviated versions of the SCDI could be designed for more rapid, scaled-up detection, although the diagnostic accuracy of such surveys needs to be further validated.

## 5. Strengths and Limitations

This review’s strength lies in its focus on psychiatrist-administered interviews for diagnosis, minimizing the subjectivity inherent in self-reported measures used by other analyses. However, significant heterogeneity persists due to variations in study populations. Subgroup analyses explored the influence of certain demographic factors (e.g., MI subtype, age, sex) on the prevalence of psychiatric conditions. Due to limitations in PTSD data, subgroup analysis for PTSD was excluded. The observational nature and potential bidirectionality between mental health conditions and AMI in all studies limit causal inference. Further research is needed to study potential interventions to reduce the risk of mental health disorders after AMI and their impact on adverse outcomes post-AMI.

## 6. Future Extension

The next steps should include longitudinal studies that track the long-term psychological outcomes of AMI patients, focusing on the effectiveness of specific interventions. There is also a need for more research on the role of pre-existing conditions, the impact of different types of AMI, and the influence of healthcare systems on psychological outcomes. Developing standardized guidelines for mental health care in post-AMI patients would also be a valuable contribution.

## 7. Conclusions

Post-AMI survivors exhibit an increased risk of psychiatric conditions such as depression, anxiety, and PTSD, which can have deleterious psychological and physical health impacts on recovery outcomes—fueling a vicious cycle between worsening chronic disease management and mental well-being. To address this, prompt screening of at-risk populations to offer early psychological support is crucial to prevent post-AMI psychiatric complications, improving the quality of life of cardiac event survivors in the long run.

## Data Availability

The original contributions presented in this study are included in the article/Supplementary Materials. Further inquiries can be directed to the corresponding author.

## Supplementary Materials

The following supporting information can be downloaded at: https://www.mdpi.com/article/10.3390/jcm14061786/s1, Table S1: Characteristics of included studies, Table S2: Newcastle-Ottawa quality assessment scale for cohort and case-control studies, Table S3: Subgroup analysis for the prevalence of depression, Table S4: Subgroup analysis for the prevalence of anxiety.
