# Supplementary material for: Prevalence of Depression, Anxiety and Post-Traumatic Stress Disorder (PTSD) After Acute Myocardial Infarction: A Systematic Review and Meta-Analysis
Source: J Clin Med. 2025 Mar 7;14(6):1786. doi: 10.3390/jcm14061786 (PMC11943088; doi:10.3390/jcm14061786)
Supplement: Supplementary file 1 [file jcm-14-01786-s001.zip › Search strategy.pdf]

Search Strat:
On Pubmed, Embase, PsycInfo

((Major Depression[Title/Abstract] OR (Depressive Disorder, Major[MeSH Terms]) OR (Depressive Disorder[MeSH Terms])) OR ((Anxiety[MeSH Terms]) OR (Angst[Title/Abstract] OR Anxiousness[Title/Abstract]))) AND ((Myocardial Infarction[MeSH Terms]) OR (Myocardial Infarct*[Title/Abstract]) OR (Acute Myocardial Infarct*[Title/Abstract]) OR (Heart Attack[Title/Abstract]))
