# Supplementary material for: Prevalence of Depression, Anxiety and Post-Traumatic Stress Disorder (PTSD) After Acute Myocardial Infarction: A Systematic Review and Meta-Analysis
Source: J Clin Med. 2025 Mar 7;14(6):1786. doi: 10.3390/jcm14061786 (PMC11943088; doi:10.3390/jcm14061786)
Supplement: Supplementary file 1 [file jcm-14-01786-s001.zip › Tables.pdf]

Table S1: Characteristics of included studies

First Author	Year	Country of population	Sample Size (N)	Diagnostic tool	Follow up timeframe (months)	Mean Age (SD)	% Male	% Married	% Smoker	% Diabetes Mellitus	% Hypertension	% 1st time MI	% Killip class I	% Hyperlipidaemia	% with Pre-existing mental illness	% Living alone
**S. J. Schleifer**	1989	USA	283	DSM-III-R	3-6	63.7 (0.7)	64	55			5	73			N.A.	
**A. W. Forrester**	1992	USA	129	ICD-9	<3	59 (15)	73.64	55.04				63			0	
**F. Lesperance**	1993	Canada	222	DSM-III	<3, 3-6	59.6	78		39.6		36.1		78.83		0	
**J. Romanelli**	2002	USA	153	DSM-III	<3	74.5	55.6			35.90	69.30	64.05	50.32	58.16	N.A.	
**I. Aben**	2003	Netherlands	200	DSM-IV	<3, 3-6, 6-12, >12	60 (10.8)	77					100			12	18
**L. L. Watkins**	2003	USA	2481	DSM-IV	<3	61 (13)	56	52	42.2	33.1	-	73	76.98	62.99	N.A.	
**F. Lespérance**	2004	Canada	481	DSM-IV	<3	60	81.1								16.84	
**J. J. Strik**	2004	Netherlands	206	DSM-IV-R	<3	59 (10.6)	75.72		14.4			100		30.58	18.93	18
**L. H. Powell**	2005	USA	847	DSM-IV	<3	64 (12.5)	54	52	28	43	66	63		64	N.A.	
**T. Spijkerman**	2005	Netherlands	468	ICD-10	<3	59.6 (12.2)			50.6				83.36		0	14.1
**J. Denollet**	2006	Netherlands	176	DSM-IV	<3	60.1 (10.7)	76.14								N.A.	
**J. P. van Melle**	2007	Netherlands	122	ICD-10	>12	57.5	76		54	14	35			84	0	
**K. Parakh**	2008	USA	284	DSM-III	<3, >12	64(13)	57.04		29.23	35.56	66.2			61.61	N.A.	21.4
**E. A. Kuhl**	2009	USA	278	DSM-III-R	<3, 3-6		57		30	35	66		60	61	N.A.	21
**R. Hari**	2010	Switzerland	274	DSM-IV	<3, >12	60	84.30%									
**M. Agarwal**	2011	India	101	DSM-IV-TR	<3	51.96(8.6)	85.14	84.16	38.61	30.69	37.62			36.63	0	
**R. L. Reese**	2011	USA	766	DSM-IV	3-6	58.2(11.8)	60.31	67.23	32.38	27.02	55.09			54.04		
**A. M. Roest**	2012	Netherlands	438	CIDI	3-6	59.5(11)	80.8		47.49	9.36	27.63		96.58	34.47	N.A.	14

**D. Edmondson**	2012	USA	420	DSM-IV	<3	63 (11)	69								
**S. Sarkar**	2012	India	103	MINI	3-6	54.6(9.35)	91.3		59.20%	24.30%	38.80%	79.61		41.74	25.24
**B. B. Annagür**	2015	Turkey	116	DSM-IV	3-6	55.19 (10.9)	88.8	94.8	19.83	23.28	45.69				N.A.
**H. P. Feng**	2016	Taiwan	1396	ICD-9	>12	65.99	63.25	nil	nil	14.11	18.98			6.23	0
**A. Baranyi**	2021	Austria	114	DSM-V	<3, 6-12	59.9 (11.48)	84.2	67.5	34.2	16.67	89.5	89.47	77.19	51.75	

DSM - Diagnostic and Statistical Manual of Mental Disorders; R - Revision; TR - Test Revision
CIDI - Composite International Diagnostic Interview
MINI - Mini International Neuropsychiatric Interview

Table S2: Newcastle-Ottawa quality assessment scale for cohort and case-control studies

Study	Selection				Comparability	Outcome			
	Representativeness of exposed cohort	Selection of external control	Ascertainment of exposure	Outcome of interest not present at the start of the study	Comparability of cohort on the basis of design or analysis	Assessment of outcome	Sufficient follow-up time	Adequacy of follow-up	Total * (out of 9)
S.J. Schleifer, 1989	*	*	*	*	**	*	*	0	8
A.W. Forrester, 1992	*	*	*	0	**	*	0	0	6
F. Lesperance, 1993	*	*	*	0	**	*	0	*	7
J. Romanelli, 2002	*	*	*	*	*	0	*	0	6
I. Aben, 2003	*	*	*	*	**	*	*	*	9
L.L. Watkins, 2003	*	*	*	0	**	*	*	*	9
F. Lesperance, 2004	*	*	*	0	**	*	*	*	8
J.J. Strik, 2004	0	*	*	*	**	*	*	*	8
L.H.Powell, 2005	*	*	*	0	**	*	*	*	8
T. Spijkerman, 2005	*	*	*	*	**	*	*	*	8
J. Denollet, 2006	*	*	*	0	*	*	*	*	7
J.P. van Melle, 2007	*	*	*	*	**	*	*	*	9
K. Parakh, 2008	*	*	*	*	**	*	*	*	9
E. A. Kuhl, 2009	*	*	*	*	**	*	*	0	8

R. Hari, 2010	*	*	*	0	**	*	*	*	8
M. Agarwal, 2011	*	*	*	*	**	*	*	*	9
R.L. Reese, 2011	*	*	*	*	**	*	*	*	9
A.M. Roest, 2012	*	*	*	0	**	*	*	*	8
D. Edmondson, 2012	*	*	*	*	**	*	0	0	7
S. Sarkar, 2012	*	*	*	0	**	0	*	0	6
B.B. Annagur, 2015	*	*	*	0	**	*	*	*	8
H.P. Feng, 2016	*	*	*	*	**	*	*	0	8
A. Baranyi. 2021	*	*	*	*	**	*	*	*	9

Table S3: Subgroup analysis for the prevalence of depression

Subgroup	Category	No. of Studies	No. of Subjects with depression	Sample size	Pooled prevalence (95% CI)(%)	I^2 value (%)	Chi^2 P value
**Gender**	Male	6	694	2491	22.06 (17.01; 28.10)	92.9	<0.01
	Female	6	621	1607	29.89 (21.85; 39.41)	87.6	
**Hyperlipidemia**	Yes	6	809	2341	28.96 (23.44; 35.17)	89.4	<0.01
	No	6	471	1650	19.69 (12.44; 29.69)	93.8	
**Killip Class**	I	5	917	2727	26.98 (18.84; 37.02)	93.2	<0.01
	>1		359	874	34.48 (23.76; 47.06)	87.4	
**Smoking Status**	Yes	6	199	719	25.23 (19.48; 32.00)	59.4	<0.01
	No	6	222	1328	16.85 (13.85; 20.35)	63.7	
**Living arrangement**	Lives alone	5	64	243	26.34 (21.18; 32.23)	**74.5**	0.86
	Does not live alone	5	292	1143	25.45 (20.92; 30.58)	71.8	
**Marital status**	Married	4	738	1961	28.63 (18.67; 41.20)	87.8	<0.01
	Unmarried	4	621	1491	35.44 (19.61; 55.26)	94.5	
**Diabetes Mellitus**	Yes	5	471	1527	34.29 (28.11; 41.04)	80.4	0.05
	No	5	920	2836	28.33 (20.32; 38.00)	90.7	
Hypertension	Yes	4	188	754	25.01 (21.68; 28.67)	10.3	0.02
	No	4	107	550	19.45 (16.35; 22.97)	0	
Anterior MI	Yes	3	69	237	29.07 (23.01; 35.97)	31.5	0.36
	No	3	149	580	31.07 (18.07; 47.95)	89.1	
First time AMI	Yes	3	796	2217	32.71 (27.52; 38.36)	80.8	
	No*	-	-	-	-	-	-
History of	Yes	3	211	486	57.41 (31.47; 79.82]	96.2	<0.01

Depression							
	No	3	461	967	46.66 (6.75; 91.36	99.4	<0.01

* No studies reported data on patients with repeat AMI only, therefore subgroup analysis was not performed for patients with repeat AMI only

Table S4: Subgroup analysis for the prevalence of anxiety

Subgroup	Category	No. of Studies	No. of Subjects with depression	Without Depression	Sample size	Pooled prevalence (95% CI)(%)	I^2 value (%)	Chi-squared P value
Gender	Male	3	87	614	701	14.33 [11.76;17.35]	97.2	<0.01
	Female	3	66	181	247	27.73 [22.41;33.76]	95.1	
Hyperlipidemia	Yes	2	87	277	364	27.11 [22.52;32.23]	97.9	<0.01
	No	2	56	399	455	14.17 [11.07; 17.97]	97.9	
Killip Class	I	3	112	898	1010	11.34 [3.11; 33.75]	98.5	<0.01
	>1	3	55	89	144	24.48 [9.62; 49.67]	76.5	
Smoking Status	Yes	3	70	429	499	14.74 [3.94;42.19]	97.5	<0.01
	No	3	97	558	655	9.95 [2.48; 32.39]	97.9	
Diabetes Mellitus	Yes	3	55	124	179	18.79 [6.10; 45.20]	92.3	<0.01
	No	3	112	863	975	10.91 [3.03; 32.41]	98.4	
Hypertension	Yes	2	93	252	345	30.49 [25.58; 35.89]	97.2	<0.01
	No	2	50	424	474	12.16 [9.34;15.69]	97.0	
